# Mesenchymal stem cells attenuate blood-brain barrier leakage after cerebral ischemia in mice

**DOI:** 10.1186/s12974-018-1153-1

**Published:** 2018-05-03

**Authors:** Zhuo Cheng, Liping Wang, Meijie Qu, Huaibin Liang, Wanlu Li, Yongfang Li, Lidong Deng, Zhijun Zhang, Guo-Yuan Yang

**Affiliations:** 1 School of Biomedical Engineering and Shanghai Jiao Tong University affiliated sixth people’s hospital, Shanghai Jiao Tong University, Shanghai, 200000 China; 2 Department of Neurology, Ruijin Hospital, School of Medicine, Shanghai Jiao Tong University, Shanghai, 200025 China

**Keywords:** Blood-brain barrier, ICAM-1, Inflammation, Matrixmetallo-proteinase-9, Ischemia, Mesenchymal stem cell

## Abstract

**Background:**

Ischemic stroke induced matrixmetallo-proteinase-9 (MMP-9) upregulation, which increased blood-brain barrier permeability. Studies demonstrated that mesenchymal stem cell therapy protected blood-brain barrier disruption from several cerebrovascular diseases. However, the underlying mechanism was largely unknown. We therefore hypothesized that mesenchymal stem cells reduced blood-brain barrier destruction by inhibiting matrixmetallo-proteinase-9 and it was related to intercellular adhesion molecule-1 (ICAM-1).

**Methods:**

Adult ICR male mice (*n* = 118) underwent 90-min middle cerebral artery occlusion and received 2 × 10^5^ mesenchymal stem cell transplantation. Neurobehavioral outcome, infarct volume, and blood-brain barrier permeability were measured after ischemia. The relationship between myeloperoxidase (MPO) activity and ICAM-1 release was further determined.

**Results:**

We found that intracranial injection of mesenchymal stem cells reduced infarct volume and improved behavioral function in experimental stroke models (*p* < 0.05). IgG leakage, tight junction protein loss, and inflammatory cytokines IL-1β, IL-6, and TNF-α reduced in mesenchymal stem cell-treated mice compared to the control group following ischemia (*p* < 0.05). After transplantation, MMP-9 was decreased in protein and activity levels as compared with controls (*p* < 0.05). Furthermore, myeloperoxidase-positive cells and myeloperoxidase activity were decreased in mesenchymal stem cell-treated mice (*p* < 0.05).

**Conclusion:**

The results showed that mesenchymal stem cell therapy attenuated blood-brain barrier disruption in mice after ischemia. Mesenchymal stem cells attenuated the upward trend of MMP-9 and potentially via downregulating ICAM-1 in endothelial cells. Adenosine 5′-monophosphate (AMP)-activated protein kinase (AMPK) pathway may influence MMP-9 expression of neutrophils and resident cells, and ICAM-1 acted as a key factor in the paracrine actions of mesenchymal stem cell.

## Background

Ischemic stroke has been identified as the second leading cause of disability and death behind ischemic heart disease worldwide [1]. In China, there are 2.4 million new strokes and 1.1 million stroke-related deaths each year [2]. Reducing high mortality and morbidity could therefore benefit stroke patients, family caregivers, and society in burden alleviation.

Matrixmetallo-proteinases are a family of zinc and calcium-dependent endopeptidases, which is capable to degrade all components of extracellular matrix (ECM) including laminin, collagen, and fibronectin surrounding the blood-brain barrier (BBB) [3]. BBB leakage is a key issue in the injury cascade, which exacerbates ischemic brain injury. Through peripheral immune cells into the brain to enhance the neuroinflammatory response, vasogenic edema comes to hasten [4, 5]. Clinical and experimental studies demonstrated that matrixmetallo-proteinase (MMP-2, MMP-3, MMP-7, or MMP-9) was activated and upregulated after ischemic stroke [6–10]. MMP-2-induced early BBB disruption is deemed reversible since the tight junction components loosen and they remain in the endothelial cleft. Thus, BBB can be reassembled. In contrast, MMP-9 is markedly elevated in the late phase of BBB disruption, which occurs at 24–72 h after ischemic stroke [11]. MMP-9 is a 92-kDa type IV collagenase, which is also called gelatinase B. MMP-9 breaks tight junction proteins (e.g., occludin and claudin-5) and basal lamina proteins (e.g., fibronectin, laminin, collagen), resulting in barrier disruption in physiological process. It is noted that inhibition of MMP-9 provided robust protection against the BBB disruption [12], suggesting that MMP-9 was the dominant protease acting at the BBB following ischemic stroke [13].

In brain, MMPs are expressed by various cell types including resident cells such as endothelial cells, microglia, neurons, astrocytes, and infiltrated inflammatory cells during ischemic stroke [14, 15]. Neutrophils are the main inflammatory cell type that responded to the inflammatory stimulus following ischemic stroke [16]. It is interested that myeloperoxidase, a major component of neutrophil azurophilic granules, is MMP-9 postive, suggesting that neutrophils are the main source of MMP-9 following ischemic stroke [17]. Transmigration of neutrophils was thought to be reliant.

Mesenchymal stem cells (MSCs), a subset of non-hematopoietic stem cells residing in bone marrow, support the growth and differentiation of hematopoietic stem cells and possibly repopulate stem cells in other tissues [18]. Systemic administration of MSCs after cerebral infarction could reduce infarct volume and improve behavioral function in experimental stroke models [19, 20]. MSCs exhibit potent immunosuppressive activity [21]. Meanwhile, they have a potential to maintain BBB integrity [22]. Our previous study showed that MSCs could protect BBB integrity by reducing the astrocyte apoptosis after ischemic attack, which was due to the attenuation of inflammatory response and downregulation of aquaporin-4 expression [22]. However, limited studies focused on the effect of MSCs on MMP-9 activity via transmigration of neutrophils.

In this research, we explored (1) whether MSC therapy attenuates BBB breakdown and reduces inflammatory response following transient MCAO in mice, (2) whether the effect of protection related to MMP-9 and how MSCs contribute to the attenuation of MMP-9 increase during transient MCAO in mice, and (3) what molecular signaling pathways involve in MSC therapy.

## Methods

### Experimental design

Animal protocol was approved by the Institutional Animal Care and Use Committee of Shanghai Jiao Tong University, Shanghai, China. All animal procedures were performed to minimize pain or discomfort in accordance with current protocols. Adult male ICR mice (*n* = 118) weighing 30–32 g were divided into four groups: (1) MSC treated (*n* = 36), (2) PBS treated (*n* = 55), (3) MMP-9 inhibitor (SB-3CT, *n* = 12), and (4) sham (*n* = 15) group. At 1 and 3 days following transient middle cerebral artery occlusion (tMCAO), neurological severity score was performed before mice were sacrificed. Then, samples were collected for further study.

The oxygen-glucose deprivation (OGD) experiment was performed using four groups differentiated as follows: (1) bEND.3 cells (mouse brain microvascular endothelial cells, endothelialpolyoma middle T antigen transformed from cerebral cortex, American Type Culture Collection (ATCC)) alone, (2) bEND.3 cells co-cultured with MSCs, (3) bEND.3 cells cultured with condition medium (CM) of MSCs, and (4) bEND.3 co-cultured with MSCs plus compound C (Sigma, St. Louis, MO). bEND.3 cells alone without oxygen-glucose deprivation was the control (ctrl).

### MSC isolation and identification

Bone-derived MSCs (MSCs) were isolated and harvested from adult male Sprague Dawley rats (SD, Jiesijie, Co., Shanghai, China) weighing 200–250 g as previously described [23]. The cells were suspended in Dulbecco’s modified Eagle’s medium (DMEM; Gibco Laboratories, Grand Island, NY) with 10% fetal bovine serum (FBS; Life Technologies, Carlsbad, CA) and incubated at 37 °C with 5% CO_2_. Identification was performed by flow cytometry (BD Biosciences, Mississauga, ON) following the instructions. Cells were incubated in 100 μl PBS with CD29-APC (1:100 dilution, BD Biosciences, Mississauga, ON), CD90-cy5.5 (1:100 dilution, BD Biosciences), CD31-PE (1:100 dilution, eBioscience, San Diego, CA), FITC-CD45 (1:100 dilution, eBioscience), and their isotype control antibodies for 20 min on ice. After three washes with PBS, the cells were suspended in 300 μl of PBS and analyzed in fluorescence-activated cell sorter (FACS) instrument (BD Biosciences).

### Transient middle cerebral artery occlusion (MCAO)

After anesthetizing mice with ketamine/xylazine (100 mg/10 mg/kg, Sigma, St. Louis, MO) intraperitoneally, the occlusion of middle cerebral artery (MCA) was achieved by inserting a silicone-coated 6-0 nylon suture (Covidien, Saint Louis, MA) from the incision on left external carotid artery (ECA) with an advancement of 9–10 mm toward MCA. Successful occlusion of MCA was confirmed using a laser Doppler flowmetry (Moor Instruments, Devon, UK) as a decline in the regional blood flow by more than 80% compared to the contralateral hemisphere. The suture was withdrawn completely, and reperfusion was achieved after 90 min of transient MCAO. The mortality in our study was less than 10%.

### Oxygen-glucose deprivation

bEND.3 cells were seeded in six-well plates and incubated with FBS-free condition medium (Gibco) at 5% CO_2_ and 95% N_2_ atmosphere using an airtight chamber for 6 h followed by re-oxygenation as previously described [24].

### Neurobehavioral assessments

Neurobehavioral assessments were conducted by an experiment partner who was blind to the treatment conditions. For neurological function assessment, a modified Neurological Severity Scores (mNSS) ranging from 0 to 14 score was adopted, which included raising the mouse by the tail (0 to 3), walking on the floor (0 to 3), beam balance tests (0 to 6), and the response absence (0 to 2).

### MSC transplantation and labeling

For cellular tracking after transplantation, cells were labeled with carboxyfluorescein diacetate-succinimidyl ester (CFDA-SE, Beyotime, Shanghai, China). MSCs were resuspended in 1 mM CFDA dye at 37 °C for 20 min. The animals received stereotaxic transplantation within 15 min after reperfusion via a 10-ml Hamilton syringe (Hamilton, Bonaduz, Switzerland) injection. The transplant coordinate was 2 mm lateral to the sagittal suture and 1 mm posterior to the coronal suture and 3 mm under the dura which is in peri-ischemic area. We injected MSC suspension with 1 × 10^5^ cells/5 μl potassium phosphate (PBS) buffer at a rate of 0.5 μl/min.

As a positive control, 2-[[(4-phenoxyphenyl) sulfonyl]methyl]-thiirane (SB-3CT; Sigma), an inhibitor of MMP-9, was also used. It was diluted in 10% dimethylsulfoxide/ 90% NS and was injected intraperitoneally at 10 mg/kg per day for 3 consecutive days beginning from days 1 to 3 after ischemia.

### Infarct volume measurement

Infarct volume was measured using Cresyl Violet acetate (Sigma) staining. Sections (200 μm apart) were obtained from all brain tissue including infarct area. The ischemic area of each section was depicted by image and measured by *Image J* (NIH, Bethesda, MD, https://imagej.nih.gov/ij/). The contralateral area minus the normal area of the ipsilateral hemisphere was recorded as the infarct area ΔS. The infarction area of two adjacent pieces are denoted as ΔS1 and ΔS2; the volume of infarction (*V*) for two adjacent cerebral volume is one third of thickness (*H*) × (ΔS1 + ΔS2 + √(S1 × S2)); the thickness (*H*) = 0.2 mm. Then, sum all cerebral infarction volume of two adjacent brain tissues.

### Immunohistochemistry

Frozen sections were fixed with methanol for 10 min and incubated in 0.3% Triton X-100 solution for 10 min and blocked with 10% bovine serum albumin (BSA) for 1 h. After blocking, the sections were incubated with primary antibodies against ZO-1 (1:200, Invitrogen, Carlsbad, CA), occuldin (1:200, Invitrogen), claudin-5 (1:200, Invitrogen), and CD31 (1:200; R&D Systems, Minneapolis, MN) overnight at 4 °C under humidified condition. After washing with PBS, sections were incubated with secondary antibody for 2 h at 37 °C to perform immunofluorescence. After closure, get observation under confocal microscope (Leica, Solms, Germany).

DAB staining for IgG and myeloperoxidase (MPO): brain sections were incubated in 0.3% H_2_O_2_ in methanol for 30 min. The primary antibody MPO (1:300 dilution, R&D Systems) were incubated overnight at 4 °C and incubated with Universal ABC Kit (Vector Laboratories). The reaction product was visualized using a DAB peroxidase substrate (Vector Laboratories, Burlingame, CA). Sections were analyzed using bright-field microscopy (Leica). Mean optical density was measured using *Image-Pro Plus* software (Media Cybernetics, Bethesda, MD, www.mediacy.com). The value of integrated optical density (IOD) after optical density correction and the area of interested was used for the quantitative analysis of IgG.

### Western blot analysis

Tissue from peri-ischemic areas was placed in RIPA Lysate (Millipore, Bedford, MA) with protease inhibitor cocktail (Thermo), phosphatase inhibitors (Thermo), and phenylmethyl sulfonyl fluoride (PMSF, Thermo) in ice for 30 min and was centrifuged at 12000 rpm for 15 min at 4 °C. The supernatant was obtained from the centrifuged mixture and stored at − 80 °C. The protein concentration of stored samples was measured using the BCA Protein Assay Kit (Thermo).

The samples were subjected to SDS-polyacrylamide gel electrophoresis and transferred to filter membrane. The membranes were blocked with 5% non-fat milk and incubated with primary antibody MMP-9 (1:1000 dilution, Millipore), ICAM-1 (1:1000 dilution, R&D Systems), and AMPK/p-AMPK (1:1000 dilution, Cell Signaling Technology, Beverly, MA) overnight. β-Actin (1:1000 dilution, Cell Signaling Technology) was employed as the loading control. The blots were incubated with the appropriate horseradish peroxidase (HRP)-conjugated secondary antibody after washing with Tris-buffered saline. Immunoblots were detected using an enhanced chemiluminescence (ECL) kit (FD Technology, Shanghai, China) and calculated using *Image J* software (NIH, Bethesda, MD).

### Real-time PCR

Total RNA extraction and real-time PCR were performed according to the manufacturer’s instructions. Total RNA was extracted from tissues around the lesional sites 1 and 3 days after transient MCAO using Trizol reagent (Invitrogen). Total RNA was reverse transcribed to cDNA with Zymoscript first-strand cDNA synthesis kit (zymo tool, Shanghai, China). Gene transcription was detected by real-time PCR in a 7900HT sequence detection system (Applied Biosystems, Foster City, CA) using specific primers designed from known sequences. GAPDH (Cell Signaling Technology) was used as an endogenous control. Sequence-specific primers for IL-1β, IL-6, TNF-α, and GADPH were showed as follows:

**Table:** 

Gene	Forward primer (5′–3′)	Reverse primer (5′–3′)	bp
IL-1β	TCTATACCACTTCACAAGTCGGA	GAATTGCCATTGCACAACTCTTT	88
IL-6	GCAACTGTTCCTGAACTCAACT	ATCTTTTGGGGTCCGTCAACT	89
TNF-α	GGAACACGTCGTGGGATAATG	GGCAGACTTTGGATGCTTCTT	213
GADPH	AGGTCGGTGTGAACGGATTTG	TGTAGACCATGTAGTTGAGGTCA	123

### MPO activity assay

MPO activity assay was performed as described previously [24]. In brief, 10 μl brain protein of which had diluted as 1:10 from ipsilateral hemisphere was added to 180 μl of work solution containing 2 mmol/l O-dianisidin-dihydrochloride (Sigma) dissolved in 50 mmol/l PBS (pH = 6). Before measurement, 10 μl of 100 mmol/l H_2_O_2_ was added. Changes in absorbance at 460 nm over 10 min were measured.

### Zymography

Dilute protein extracts with Zymogram sample buffer (Bio-Rad, Hercules, CA). Use 10% SDS-polyacrylamide gels containing 1% gelatin (Sangon Biotech, Shanghai, China) to run 150 V for about 1 h; float the gels in 2.5% Triton X-100 to remove the SDS. Incubate the gels for 72 h at 37 °C in 1× LSCB solution (10×: 0.5 M Tris Base, pH 7.6, 2.0 M NaCl, 0.05 M CaCl_2_, 0.2% (*w*/*v*) Brij-35). Stain the gels for 1 h in coomassie stain (Coomassie Brilliant Blue, 30% methanol, 10% acetic acid). Store the gels in 10% acetic acid for destain gels for photography and densitometric analysis which measured the gelatinolytic activities.

### Statistical analysis

Results were presented as mean ± SD. Statistical analysis was evaluated by *GraphPad Prism 5* software (San Diego, CA, https://www.graphpad.com/). For comparison between the two groups, statistical significance was determined through Student’s *t* test. For comparison among multiple groups, statistical significance was evaluated using one-way ANOVA followed by a Student-Newman-Keuls test. *p* < 0.05 was considered statistically significant.

## Results

### MSC isolation, identification, and injection

**Fig. 1:**
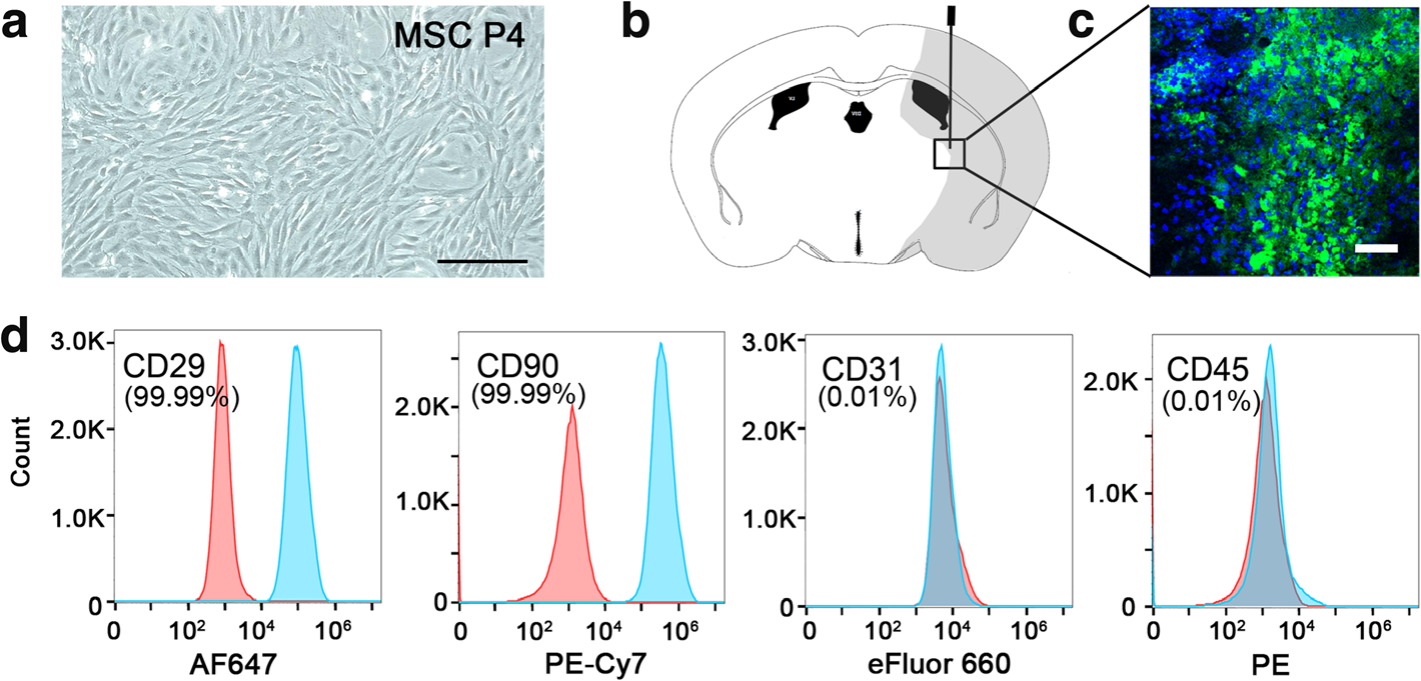
Bone-derived mesenchymal stem cell (MSC) isolation, identification, and injection. **a** Morphology of MSCs in cell culture. Cultured cells showed typically spindle-shaped morphology under phase-contrast microscopy. **b** Schematic diagram of cell injection into the striatum of the brain and the survival of MSCs after injection. **c** Green fluorescent (CFDA SE dye) cells were located in the ischemic hemisphere after 3 days of injection. Scale bar = 300 μm. **d** Cytometry analysis depicted that 99.99^+^% cultured cells were positive for CD29 and CD90 and negative for CD31 and CD45

MSCs were isolated from the femoral and tibial bone marrow of adult male SD rats. Cultured cells showed typically spindle-shaped morphology under phase-contrast microscopy (Fig. 1a). Cytometry analysis depicted that 99.99^+^% cultured cells were positive for CD29 and CD90. By contrast, CD31 and CD45 were expressed in only 0.1% of total cells (Fig. 1d). Green fluorescent (CFDA dye)-positive cells, suggesting a number of survived transplanted cells, were located in the ischemic hemisphere after 3 days of injection (Fig. 1b, c).

### MSC transplantation improved neurological outcomes and reduced stroke volume following transient MCAO

**Fig. 2:**
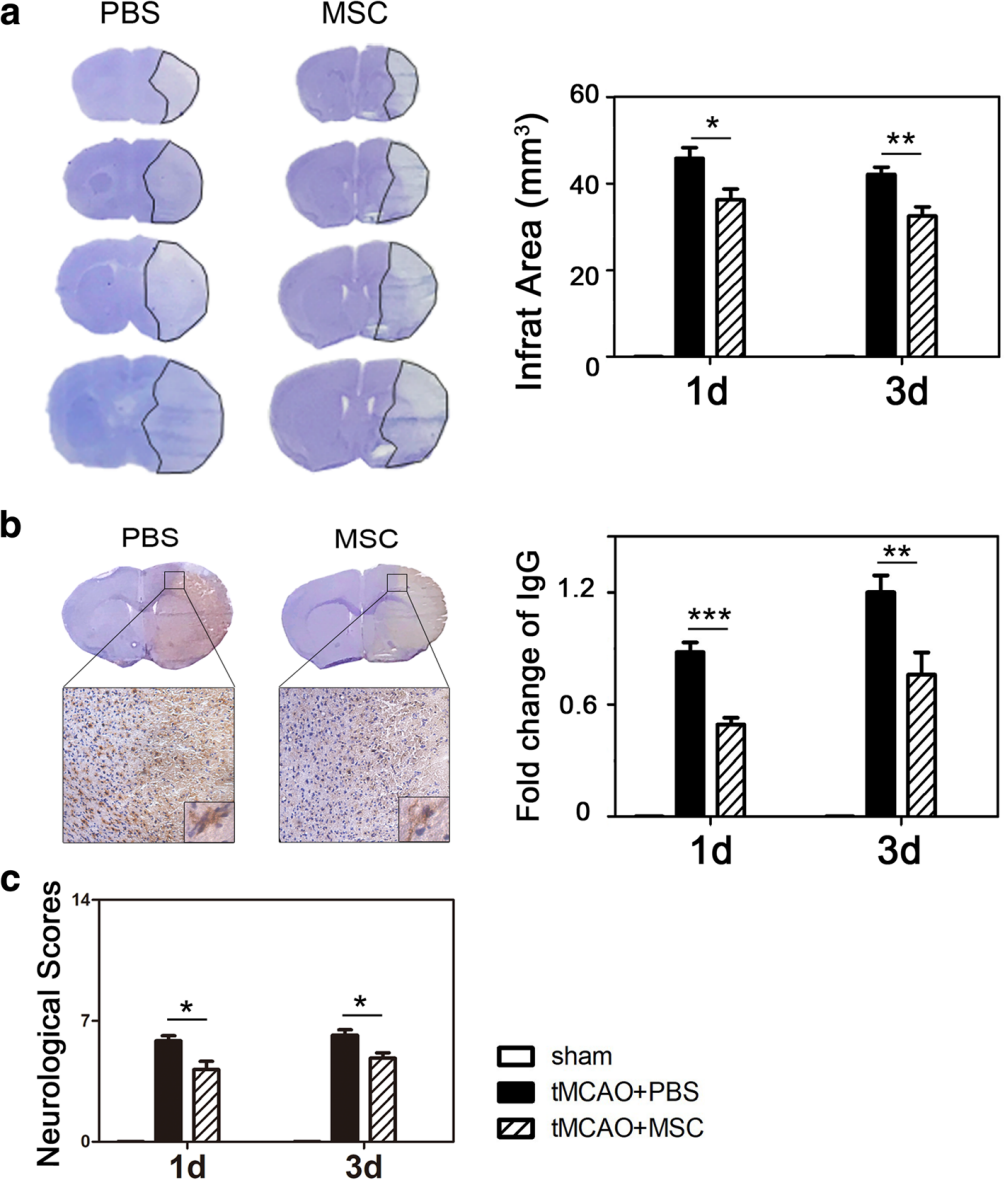
MSC transplantation improved neurological outcomes and reduced infarct volume in mice via maintaining BBB integrity following transient MCAO. **a** Photographs showed a series of coronal sections with Cresyl Violet staining following transient MCAO in the PBS- and MSC-transplanted mice. Bar graph showed the quantification of both groups. **b** IgG staining displayed that IgG protein leaked into brain tissue in PBS- and MSC-treated mice at day 3. Quantitative analysis of leaked IgG protein revealed that less IgG protein leaked into brain tissue in MSC-treated mice compared to PBS-treated mice. The images in square frames of graphs (below) were amplified as images (above), respectively. **c** MSCs improved neurological outcomes. MSCs significantly ameliorated neurological outcomes in the ischemic hemisphere at both days 1 and 3 compared with the PBS group. Data are mean ± SD, *n* = 5–6 per group. *p < 0.05, **p < 0.01, ***p < 0.001

We tested the neurological outcomes at 1 and 3 days after transient MCAO using mNSS (Fig. 2c). The results showed that the neurological deficits were reduced after MSC transplantation following 1 and 3 days of transient MCAO (*p* < 0.05). MSCs ameliorated infarct volume in the ischemic hemisphere at days 1 and 3 compared to the PBS group (*p* < 0.05) (Fig. 2a).

### MSC transplantation attenuated BBB breakdown following transient MCAO

**Fig. 3:**
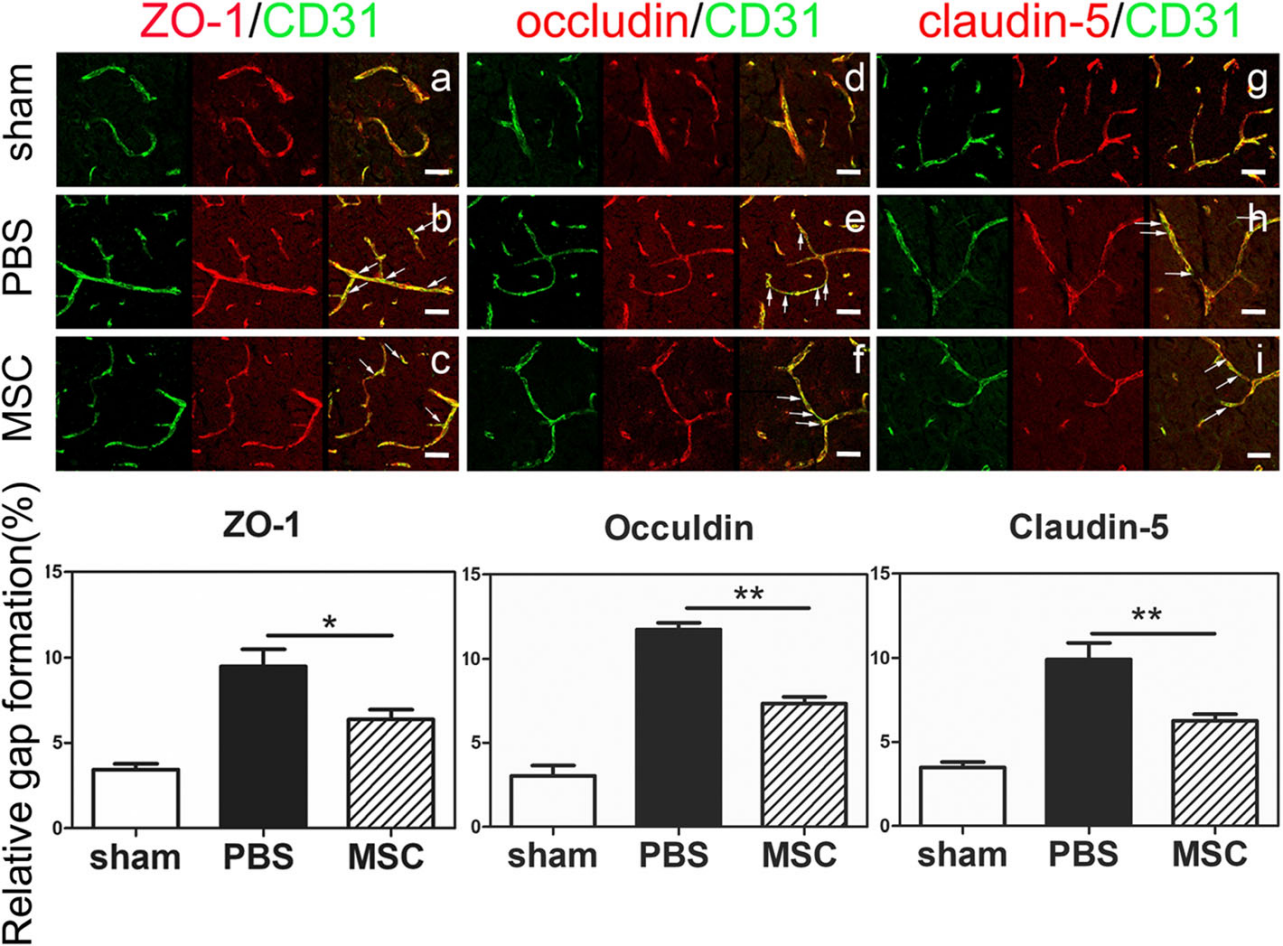
MSCs reversed gap formation of ZO-1, occludin, and claudin-5. Sections from ischemic penumbra were stained of ZO-1, occludin, and claudin-5 (red) and then co-stained with endothelial marker CD31 (green). Discontinuous labeling and gap formation (white arrows) were observed in ipsilateral brains following 3 days of tMCAO. Scale bar = 30 μm. Data are mean ± SD, *n* = 5–6 per group. *p < 0.05, **p < 0.01, ***p < 0.001

For evaluating BBB permeability, IgG staining displayed that IgG protein leaked into brain tissue after transient MCAO at days 1 and 3. Quantitative analysis revealed less IgG protein leaked into brain tissue in MSC-treated mice compared to PBS-treated mice (*p* < 0.05). MSC transplantation reduced IgG leakage at day 3 (Fig. 2b). To evaluate tight junction distribution in endothelial cells after MSC transplantation, we conducted immunostaining with specific cell markers: occludin/CD31, ZO-1/CD31, and claudin-5/CD31co-staining in sections from ischemic penumbra (Fig. 3). MSCs reversed gap formation of ZO-1, occludin, and claudin-5, which indicated that BBB integrity was protected by MSC treatment (*p* < 0.05).

### MSC therapy reduced expression of IL-1β, IL-6, and TNF-α and alleviated neutrophil infiltration following MCAO

**Fig. 4:**
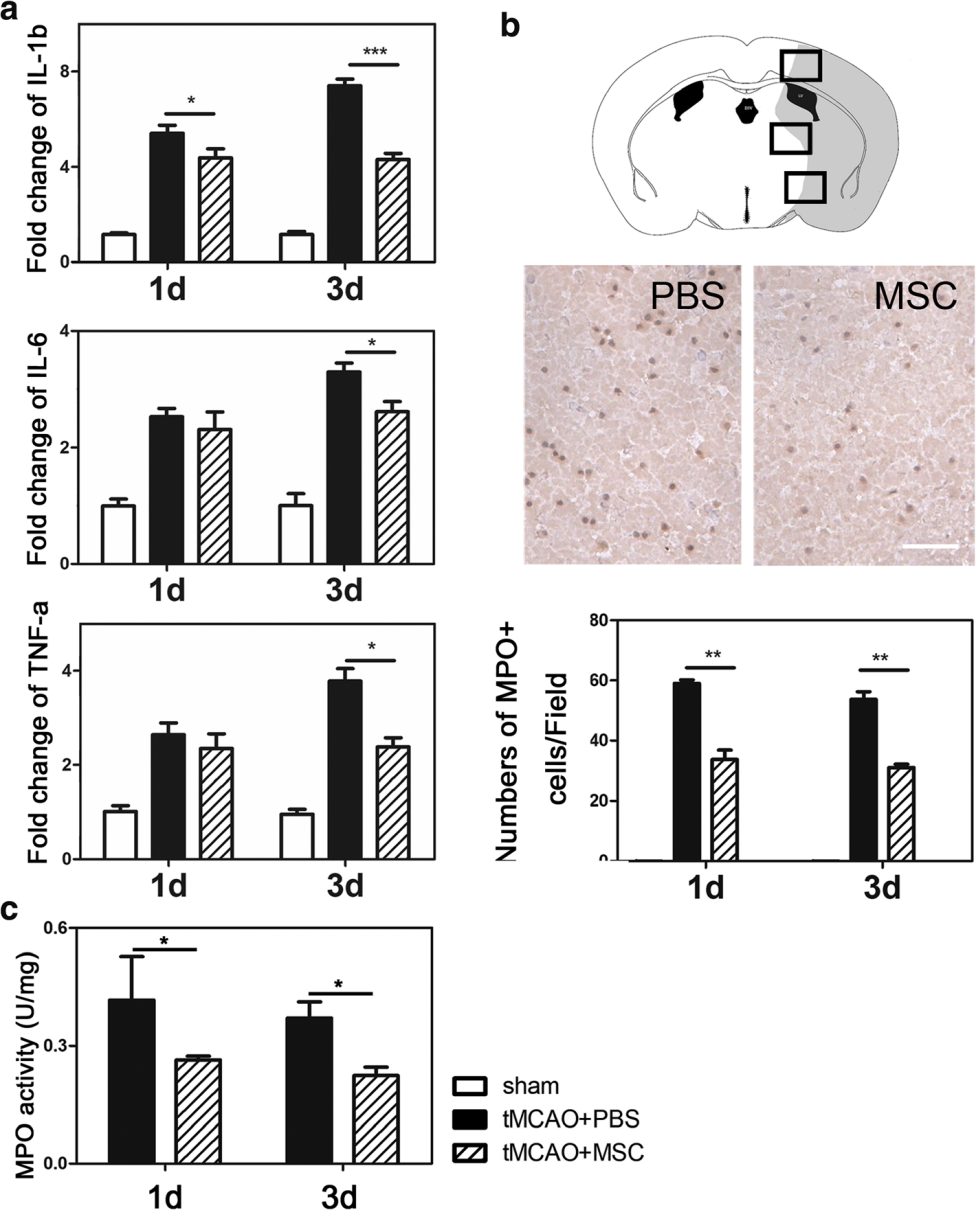
MSCs alleviated neutrophil infiltration and inflammatory cytokine expression following transient MCAO. **a** The relative mRNA expression of IL-1β, IL-6, and TNF-α normalized to GAPDH was measured at days 1 and 3 following MCAO. They were significantly decreased in the MSC-treated group compared to the PBS group at day 3; however, only IL-1β was significant at day 1. **b** MPO+ cells and their quantification in the PBS- and MSC-transplanted mice at 1 and 3 days following MCAO. In ischemic hemisphere, there were less MPO^+^ cells per field in MSC-transplanted group than PBS group, which represented less neutrophil adhesion and infiltration. Scale bar = 100 μm. **c** Bar graph showed the MPO activity in sham and PBS- and MSC-treated mice. Data are mean ± SD, *n* = 5–6 per group. *p < 0.05, **p < 0.01, ***p < 0.001

To determine whether the effect of MSCs on BBB breakdown after MCAO was involved in the immunomodulatory influence of MSCs, we examined IL-1β, IL-6, and TNF-α mRNA in mice brain (Fig. 4a). We demonstrated that IL-1β, IL-6, and TNF-α mRNA were increased at days 1 and 3 following MCAO (*p* < 0.05). Three cytokines decreased in MSC-treated group at day 3 compared to the PBS group. To investigate the neutrophil infiltration in the acute phase of cerebral ischemia, we performed DAB staining to detect MPO-positive cells (Fig. 4b). There was a decrease in MPO-positive cells at 1 and 3 days after transient MCAO in the MSC-transplanted group compared to the PBS group (*p* < 0.05). MPO activity is an indicator of neutrophil. Reduced neutrophil infiltration means reduced activity and declined function. To determine the status changes of neutrophil, MPO activity was expressed as units per milligram tissue (Fig. 4c). One unit of MPO activity represents the amount of enzyme degrading 1 μmol H_2_O_2_ per minute at 25 °C. It was noted that neutrophil infiltration was greatly reduced in the MSC-transplanted mice after transient MCAO (*p* < 0.05).

### MSCs suppressed MMP-9 upregulation after transient MCAO

**Fig. 5:**
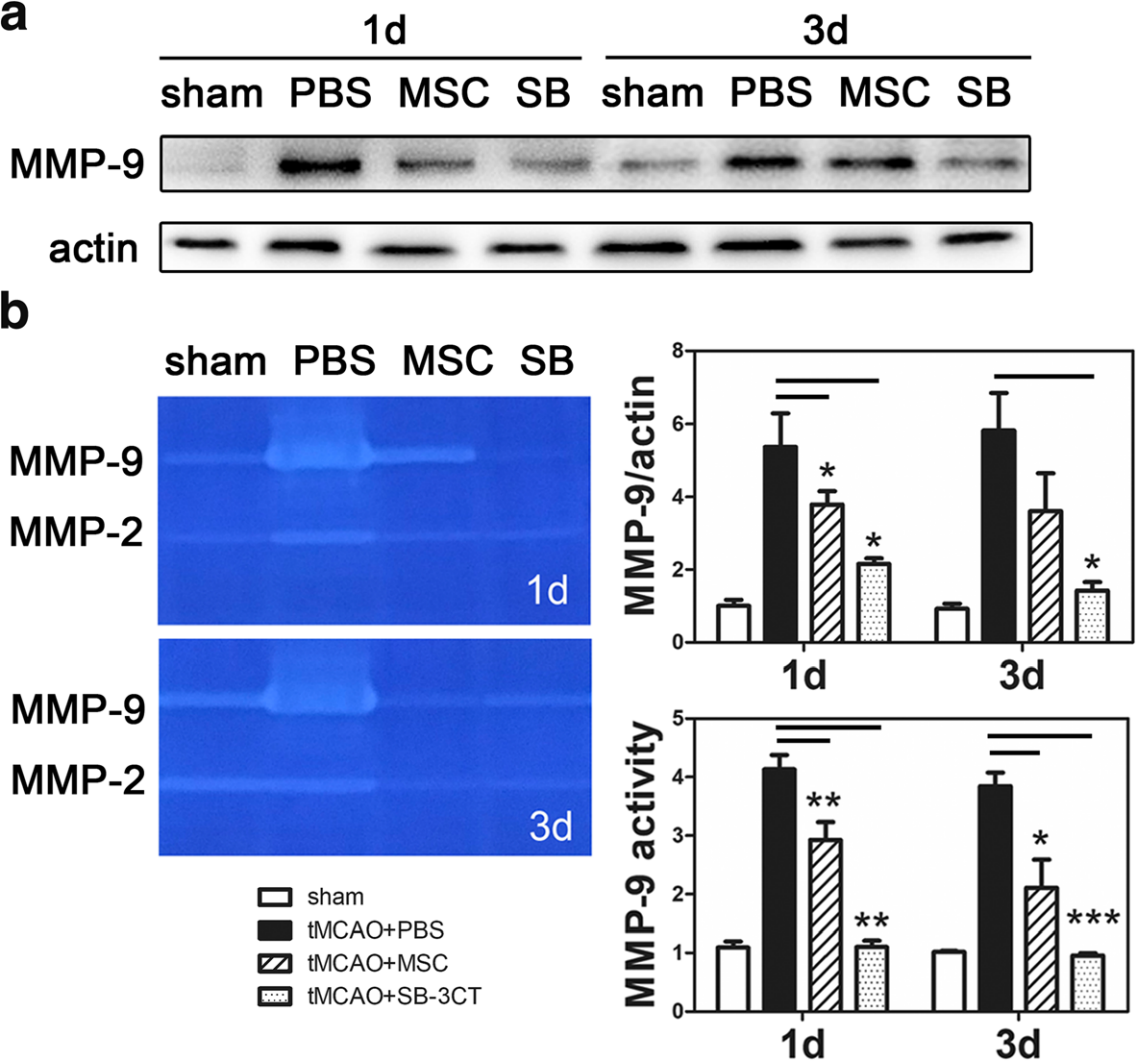
MSCs significantly inhibited MMP-9 upregulation in protein level and activity at days 1 and 3 following transient MCAO. **a** Western blot analysis of MMP-9. Treatment with MSCs downregulated the levels of MMP-9 1 and 3 days after tMCAO when compared with the PBS group. *n* = 4–6 in each time point per group. **b** Zymography and quantification of active MMP-9 levels in mice brain from four groups at 1 and 3 days after tMCAO. Data are mean ± SD. *p < 0.05, **p < 0.01, ***p < 0.001

Western blot and zymography analysis of MMP-9 were performed in the PBS, MSC-treated, and SB-3CT groups (Fig. 5). We demonstrated that ischemia-reperfusion increased MMP-9 expression and this increase was attenuated after MSC transplantation, which was similar to SB-3CT, the inhibitor of MMP-9. Brain injury-induced MMP-9 upregulation contributed to BBB leakage, but MSC transplanted lowered the MMP-9 transplanted and function which inhibited the critical BBB breakdown after ischemia (*p* < 0.05).

### MSCs downregulated ICAM-1 expression in vivo and in vitro

**Fig. 6:**
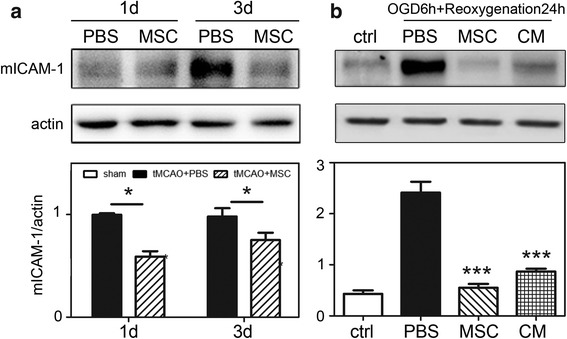
MSCs might inhibit matrix metallo-proteinase-9 upregulation via reducing the expression of endogenous ICAM-1. **a** Western blot analysis showed the expression of ICAM-1 between PBS- and MSC-treated group at days 1 and 3 following tMCAO. **b** Western blot analysis showed the expression of mICAM-1 between (1) bEND.3 cells without OGD, (2) bEND.3 treated with PBS, (3) bEND.3 cells co-cultured with MSCs after OGD, and (4) bEND.3 cells cultured with the condition medium of MSCs after OGD in vitro. *p < 0.05, **p < 0.01, ***p < 0.001

To explore the relationship between MMP-9 and ICAM-1 in vivo, we executed western blot for ICAM-1 which was related to neutrophil transmigration. Results showed the significant reduction of ICAM-1 expression in MSC-transplanted group (*p* < 0.05) (Fig. 6a). Similar result was obtained from in vitro: after OGD/re-oxygenation treatment, co-culture bEND.3 cells with MSCs displayed effective inhibition (*p* < 0.05). We further used the MSC condition medium (CM) to confirm whether it was MSC’s paracrine contributed to the ICAM-1 downregulation in cultured bEND.3 cells (Fig. 6b). The experimental results were consistent with our conjecture. ICAM-1 was expressed constituently at a low level in endothelial cells, and after OGD the expression was elevated in protein level. Both MSC condition medium and co-culture could inhibit the upregulation of ICAM-1 in bEND.3 cells. ICAM-1 acted as a key factor in the paracrine actions of MSCs.

### MSCs downregulated ICAM-1 expression which could be via AMPK signaling

**Fig. 7:**
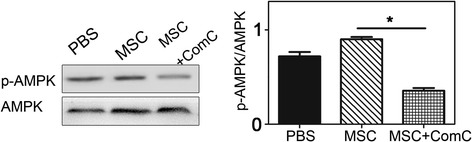
MSC transplantation promoted phosphorylation of AMPK which might reduce ICAM-1 expression in vitro. Western blot showed p-AMPK and AMPK expression and quantification in PBS- and MSC-treated and MSCs plus compound C groups at 24 h after re-oxygenation in OGD model. Bar graph showed the quantification of p-AMPK/AMPK ratio. *p < 0.05, **p < 0.01, ***p < 0.001

We performed western blot to assess the relationship between MSC-induced downregulation of ICAM-1 and the phosphorylation status of AMPK at threonine residue (Fig. 7). We analyzed the effect of MSC at a cellular level using OGD models to mimic in vivo ischemia/reperfusion injury. Results indicated that ICAM-1 was reduced in MSC-treated group compared to the PBS-treated group (*p* < 0.05). To test whether AMPK pathway was involved in MSC group after OGD, we also used a selective AMPK inhibitor, compound C to break the AMPK phosphorylation. We found that compound C reduced MSC-induced AMPK phosphorylation (*p* < 0.05). The results suggested that MSC-induced change of ICAM-1 might be AMPK dependent. Thus, we conclude that MSCs diminished the expression of ICAM-1 and potentially via an AMPK-mediated signaling pathway.

## Discussion

Our studies revealed that the beneficial effects of MSC on BBB after ischemia were due to the decrease of MMP-9 expression in neutrophil cells. These effects were mediated via an AMPK-dependent ICAM-1 downregulation potentially, thus alleviated neutrophil infiltration and inflammatory cytokines release in cerebral ischemic mice.

MMP-9 is confirmed to be a key protease interfering with BBB leakage and natural evolution of cerebral ischemia. It is produced in a prime form within cells, then activated by cleavage off the propeptide after release to extracellular space [16, 25]. In particular, MMPs are tightly regulated at both transcriptional and post-transcriptional level by transcription factors and tissue inhibitors of MMPs (TIMPs) [26]. Moreover, it has been proposed that detrimental effects of tPA beyond the 3 h of stroke onset are derived from tPA’s ability to activate MMP-9 [27]. This in turn contributes to the breakdown of BBB [28]. Certainly, MMP-9 has been the most widely studied MMP family member in BBB leakage, leukocyte infiltration, brain edema, and hemorrhage [14, 15]. Indeed, pro-MMP-9 is expressed almost exclusively in neutrophils in peripheral blood [16] and release to degrade the basal lamina of the endothelial cells and astrocytes locally at BBB [29, 30].

Several studies strongly suggest and demonstrate the implication of recruited leukocytes as a significant source of MMP-9 causing basal lamina degradation and BBB breakdown after transient ischemia [17, 31]. Neutrophils roll along and adhere to endothelium so that transmigrate through the endothelial cell barrier into the brain parenchyma to initiate the release/production of MMP-9 from resident brain cells (e.g., neurons, glial cells) to perpetuate the injury cascade [32, 33]. Increased BBB permeability induced by leukocyte-derived MMP-9 has been shown to correlate with peak neutrophil infiltration in ischemia-reperfusion injury [16, 29, 34]. In this study, MSCs affect neutrophil adhesion and transmigration which attenuates the upward trend of MMP-9 from filtrating neutrophils and resident cells. Modulating MMP-9 or neutrophil infiltration processes could be a target for future investigations to improve the present thrombolytic therapy for ischemic stroke.

Furthermore, the recruitment of leukocytes by the vascular endothelium plays a deleterious role during multistep event in the evolution of inflammatory lesions; endothelial cell adhesion molecules serve not only as docking structures but also initiate signal cascades. In addition to facilitating leukocyte adhesion, cell adhesion molecules might also contribute to the overall pro-inflammatory activation of the endothelial cells [35]. In our study, endothelial cell adhesion molecules ICAM-1 is directly involved in sustaining leukocyte adhesion. These observations support the hypothesis that attenuation of observed immunomodulatory effects of MSC may contribute to the adhesion molecule expression. ICAM-1 is required for lymphocyte adhesion and, thus, plays an important role in MSC-mediated immunosuppression [36].

AMP-activated kinase (AMPK) is a highly conserved heterotrimeric kinase that functions as a metabolic switch, thereby coordinating the cellular enzymes involved in carbohydrate and fat metabolism to enable ATP conservation and synthesis [37]. AMPK is activated by conditions that increase the AMP:ATP *ratio*, such as exercise and metabolic stress. When the AMP:ATP ratio increases, AMPK is activated by AMPK kinase, and a conformational change is induced by combining with AMP, thereby decreasing the AMP:ATP ratio by switching off ATP-consuming pathways and switching on ATP-generating pathways [37]. In this study, phosphorylation of AMPK in bEND.3 cells was upregulated in hypoxia but downregulated after co-culture with MSCs. We also found that inhibiting AMPK activation by compound C could reverse MSC-induced AMPK phosphorylation in vitro. It is generally accepted that ICAM-1 is a NF-κB-mediated gene [38]. AMPK activation may be responsible for the inhibition of NF-κB activation, so we suppose that MSC inhibits the cytokine-induced expression of pro-inflammatory and adhesion molecule genes by suppressing NF-κB activity via AMPK activation, which needs to be further studied.

MSCs have been shown to have therapeutic potential in multiple disease states characterized by vascular instability. Much of the demonstrated potential of MSCs in human disease models has been shown to be due to the production of soluble factors. Some scholars have studied in traumatic brain injury (TBI) or intracerebral hemorrhage (ICH), TIMP-1, or TIMP-3 released by MSCs could stabilize BBB integrity [39, 40]. The protective effect of MSCs on BBB was possibly invoked by increased expression of tumor necrosis factor alpha-stimulated gene-6 (TSG-6), which may suppress the activation of the NF-κB signaling pathway [41]. We had considered the effects of TIMPs secreted by MSCs could reduce MMP-9 activity in ischemic BBB initially, but our in vivo results showed that, in addition to the changes in MMP-9 vitality shown by zymography, protein levels were obviously changed. Thus, there should be other secreted substances of MSCs, which severely affected the production of MMP-9 rather TIMP. Our in vitro data showed that MSC condition medium was capable of inducing ICAM-1 overexpression in bEND.3 cells indicating that MSCs produced soluble factors to regulate the function of endothelial cells. But what MSCs secreted to decrease ICAM-1 expression in ECs was unclear at this time. In light of these findings, we suggested that secretion of certain substances MSCs attenuated the cytokine-induced expression of pro-inflammatory and adhesion molecule genes via AMPK activation. Identifying these factors is considered as the possibility of a cell-free therapeutic instead of cells. We demonstrated that ICAM-1 participation in stem cell-mediated immune-suppression would be helpful in guiding the application of clinical anti-adhesion therapies.

## Conclusions

MSCs exerted a potent regulating function on endothelial ICAM-1 expression, which consequently attenuated neutrophil infiltration, MMP-9 function, and ischemia-induced BBB disruption. We highlighted the potential relationship between immunomodulatory effect and MMP-9. Our investigation revealed that MMP-9 might associate with the infiltration of neutrophils in MSC therapy after ischemia for the first time. These strategies might provide a new insight for future therapies that aim to prevent breakdown of the BBB and eventually offer therapeutic options for stroke.

## Abbreviations

BBB: Blood-brain barrier
ICAM-1: Intercellular adhesion molecule-1
MCAO: Middle carotid artery occlusion
MMP: Matrixmetallo-proteinase-9
MSCs: Mesenchymal stem cells
OGD: Oxygen-glucose deprivation
rtPA: Recombinant tissue plasminogen activator
